# Thioridazine Enhances P62-Mediated Autophagy and Apoptosis Through Wnt/β-Catenin Signaling Pathway in Glioma Cells

**DOI:** 10.3390/ijms20030473

**Published:** 2019-01-22

**Authors:** Cheng-Wei Chu, Huey-Jiun Ko, Chia-Hua Chou, Tai-Shan Cheng, Hui-Wen Cheng, Yu-Hsin Liang, Yun-Ling Lai, Chen-Yen Lin, Chihuei Wang, Joon-Khim Loh, Jiin-Tsuey Cheng, Shean-Jaw Chiou, Chun-Li Su, Chi-Ying F. Huang, Yi-Ren Hong

**Affiliations:** 1 Graduate Institute of Medicine, College of Medicine, Kaohsiung Medical University, Kaohsiung 807, Taiwan; ashbychu@gmail.com (C.-W.C.); o870391@yahoo.com.tw (H.-J.K.); lucifer0408@hotmail.com (C.-H.C.); 4a1h0010@gmail.com (Y.-L.L.); k00511882@gmail.com (C.-Y.L.); jokhlo@kmu.edu.tw (J.-K.L.); sheanjaw@kmu.edu.tw (S.-J.C.); 2 Division of Neurosurgery, Department of Surgery, Kaohsiung Municipal Ta-Tung Hospital, Kaohsiung 801, Taiwan; 3 Department of Biotechnology and Laboratory Science in Medicine, Institute of Biopharmaceutical Sciences, National Yang-Ming University, Taipei 112, Taiwan; mountain1002@yahoo.com.tw (T.-S.C.); cornbug0425@hotmail.com (H.-W.C.); camille1988726@gmail.com (Y.-H.L.); 4 Department of Biotechnology, Kaohsiung Medical University, Kaohsiung 807, Taiwan; chwang@kmu.edu.tw; 5 Department of Neurosurgery, Kaohsiung Medical University Hospital, Kaohsiung 807, Taiwan; 6 Department of Medical Research, Kaohsiung Medical University Hospital, Kaohsiung 807, Taiwan; 7 Department of Biological Sciences, National Sun Yat-Sen University, Kaohsiung 804, Taiwan; tusya@mail.nsysu.edu.tw; 8 Department of Biochemistry & Graduate Institute of Medicine, Kaohsiung Medical University, Kaohsiung 807, Taiwan; 9 Department of Human Development and Family Studies, National Taiwan Normal University, Taipei 106, Taiwan; chunlisu@ntnu.edu.tw

**Keywords:** thioridazine, glioblastoma, Wnt/β-catenin, P62, autophagy, apoptosis

## Abstract

Thioridazine (THD) is a common phenothiazine antipsychotic drug reported to suppress growth in several types of cancer cells. We previously showed that THD acts as an antiglioblastoma and anticancer stem-like cell agent. However, the signaling pathway underlying autophagy and apoptosis induction remains unclear. THD treatment significantly induced autophagy with upregulated AMPK activity and engendered cell death with increased sub-G1 in glioblastoma multiform (GBM) cell lines. Notably, through whole gene expression screening with THD treatment, frizzled (Fzd) proteins, a family of G-protein-coupled receptors, were found, suggesting the participation of Wnt/β-catenin signaling. After THD treatment, Fzd-1 and GSK3β-S9 phosphorylation (inactivated form) was reduced to promote β-catenin degradation, which attenuated P62 inhibition. The autophagy marker LC3-II markedly increased when P62 was released from β-catenin inhibition. Additionally, the P62-dependent caspase-8 activation that induced P53-independent apoptosis was confirmed by inhibiting T-cell factor/β-catenin and autophagy flux. Moreover, treatment with THD combined with temozolomide (TMZ) engendered increased LC3-II expression and caspase-3 activity, indicating promising drug synergism. In conclusion, THD induces autophagy in GBM cells by not only upregulating AMPK activity, but also enhancing P62-mediated autophagy and apoptosis through Wnt/β-catenin signaling. Therefore, THD is a potential alternative therapeutic agent for drug repositioning in GBM.

## 1. Introduction

Glioblastoma multiforme (GBM, grade IV astrocytoma) is the most prevalent form of central nervous system (CNS) tumor. Despite recent advances in radiation and chemotherapy with temozolomide (TMZ) and cisplatin, the survival time of patients with GBM is less than 15 months after diagnosis [1]. Even with a combination of adjuvant chemoradiation therapy and surgery, the five year survival rate remains less than 10% [2]. Therefore, developing more effective therapeutic strategies, such as drug synergism of TMZ with another compound, is crucial to improve the clinical outcome of GBM treatment. Notably, treatment of GBM cells with TMZ results in autophagy and apoptosis [3,4,5].

Autophagy and apoptosis are crucial self-destructive processes that serve as internal balancing mechanisms to maintain homeostasis in eukaryotic cells. Autophagy may either involve cell death, also named type II programmed cell death, or play a prosurvival role as part of an adaptive and detoxifying process in response to sublethal stresses such as starvation, hypoxia, heat shock, and microbial pathogens [6]. Moreover, autophagy plays a critical role in the significant reduction in tumor growth [7]. During autophagy, autophagosomes engulf cytoplasmic components, resulting in the conjugation of a cytosolic form of LC3 (LC3-I) to phosphatidylethanolamine to form an LC3-phosphatidylethanolamine conjugate (LC3-II). P62 is a multifunctional adapter protein, which is implicated in both apoptotic and autophagic processes. In the autophagic degradation of ubiquitinated substrates, P62 first interacts with polyubiquitinated proteins through the ubiquitin-associated domain [8]. Additionally, P62 acts as the signaling core in orchestrating apoptosis [9]. 

The Wnt/β-catenin pathway plays a key role in GBM cells [10]. Zhang et al. found that through reprograming the expression of tumor-associated genes, the Wnt/β-catenin pathway promoted glioblastoma cancer tumorigenesis and progression [11]. Furthermore, the T-cell factor (TCF)-β-catenin complex represses P62 [10,12], which is responsible for binding to LC3, and once lipidated, is associated with phagophores and is involved in cargo recognition [10,13]. Finally, WNT signaling (involving GSK3β inhibition) activates mammalian target of rapamycin (mTOR) and protein translation [14]. 

Thioridazine (THD) is an antipsychotic medication (also referred to as a neuroleptic drug) and is widely used to treat severe psychiatric disorders. Phenothiazines (PTZ) represent a major class of antipsychotic drugs, indeed, PTZ have also been reported to induce cancer cell death and sensitize them to chemotherapy [15]. It has also been proven that PTZ has anti-cancer stem cell ability and THD was the main focus of interest [16,17,18], however, trifluoperazine [19] and chlorpromazine [20] presented similar anti-cancer stem cell activity at clinically relevant concentrations. In addition, THD could induce an increase in AMP-activated protein kinase (AMPK) activation by neutralizing a crucial regulator of cancer apoptosis and sensitizing GBM cells through the PI3K/Akt/p70S6K signaling pathway [21]. Furthermore, a study reported that THD improved sensitization of GBM cells to TMZ cytotoxicity by inducing the accumulation of LC3I/II and P62, an effect mediated by impaired late-stage autophagy [16]. 

Previous studies demonstrated the anti-glioma activity of THD through induced autophagy [16,21], but the precise autophagy-inducing pathway remains unclear. In the present study, we investigate the influence of THD on GBM as well as the underlying autophagy and apoptosis mechanisms. Our data reveal that THD induces autophagy in GBM cells through not only upregulated AMPK activity, but also enhanced P62-mediated Wnt/β-catenin signaling. 

## 2. Results

### 2.1. Both THD and THD Analogs Had Potent Effects on GBM Cell Viability

**Figure 1:**
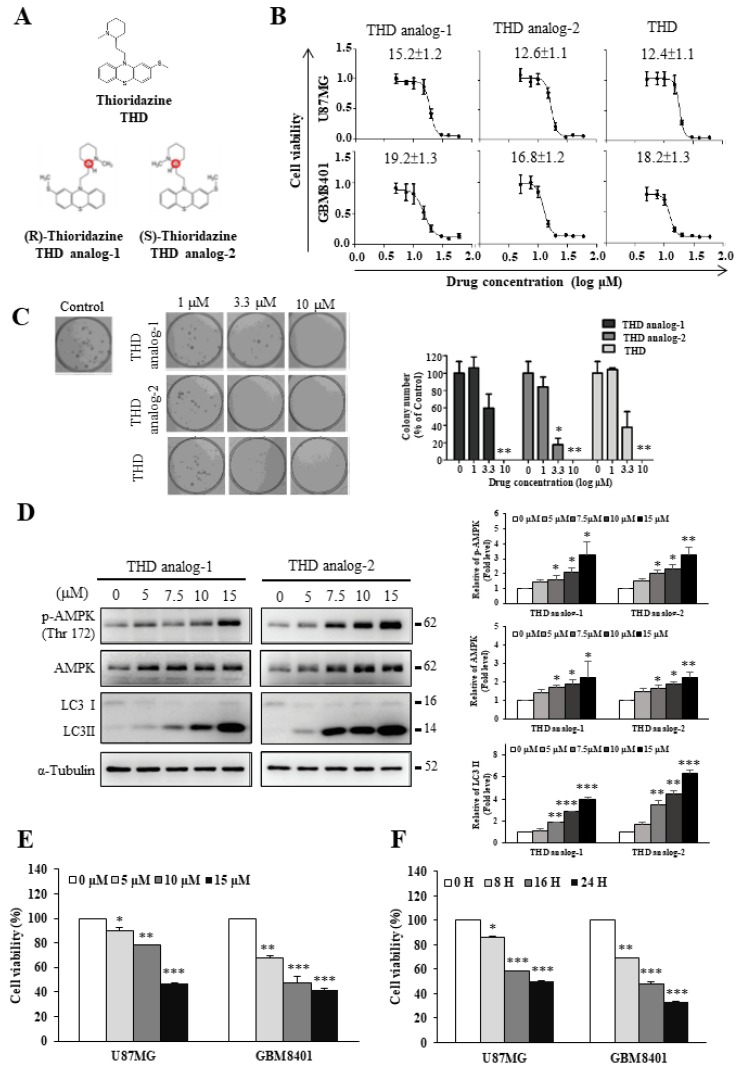
Thioridazine (THD) and THD analog treatments exerted an inhibitory effect on glioblastoma multiform (GBM) cells. (**A**) Structure of THD and enantiomers of the THD analog; (**B**) Cell viability was assessed in GBM8401 and U87MG cells by using the sulforhodamine B (SRB) assay. After 24 h drug treatments, the IC_50_ values of drugs were calculated using GraphPad Prism (THD analog-1 and analog-2 are racemic compounds). A summary of IC_50_ values is provided in the table; (**C**) Clonogenic assays were performed to assess the effect of THD and THD analogs on colony formation in GBM8401 cells. The IC_50_ values of all drugs were less than 10 µM; (**D**) GBM8401 cells were treated with different analogs of THD at 5, 7.5, 10, and 15 µM for 24 h separately. p-AMPK (Thr172), AMPK, LC3I, and LC3-II were detected using Western blotting. α-Tubulin was used as an internal control; (**E**) THD suppressed the proliferation of glioma cell lines. U87MG and GBM8401 cells were treated with various concentrations of THD for 24 h; (**F**) Cells were incubated for 8, 16, and 24 h in the presence of THD (15 µM), after which cell viability was assessed using CCK-8 assays. Bar graphs represent the mean of triplicates ± SD. * *p* < 0.05, ** *p* < 0.01, *** *p* < 0.001 compared with the control group.

THD, a drug commonly used for the treatment of schizophrenia and psychosis, is one of the few antipsychotic phenothiazine drugs with stereoisomeric forms. The structures of THD and THD analogs are presented in Figure 1A. We separated (R)-THD and (S)-THD from THD, and named as THD analog-1 and -2, respectively, in this report.

To examine whether THD and its analogs exert antitumor effects on GBM, we used the SRB and clonogenic assays to verify the cytotoxic effect of these drugs on GBM cell lines, U87MG, and GBM840. THD inhibited cell growth in the GBM cell lines in a dose-dependent manner (Figure 1B). The half maximal inhibitory concentration (IC_50_) values of THD analog-1, THD analog-2, and THD in the GBM8401 cells were 19.2 ± 1.3, 16.8 ± 1.2, and 18.2 ± 1.3 µM, respectively, and those in the U87MG cells were 15.2 ± 1.2, 12.6 ± 1.1, and 12.4 ± 1.1 µM, respectively (Figure 1B). In addition, we used the clonogenic assay, which correlated efficiently with the in vivo assay of tumorigenicity. With clonogenic assay, which represented in vivo tumorigenicity, all these drugs were effective against tumor sphere formation in the clonogenic assay of the GBM8401 cells (Figure 1C). In GBM 8401 clonogenic assay, the IC_50_ values of THD analog-1, THD analog-2, and THD were 4.4, 1.8, and 3.5 µM, respectively. These results suggested that cell viability was inhibited in the THD-treated GBM cells. To investigate the mechanisms underlying the cytotoxic effects of THD, a micro-Western assay was used to examine protein levels in the THD-treated GBM cells, and the pathway was then analyzed using the ConsensusPathDB database in our previous study [21]. Our results demonstrated the mechanisms underlying the cytocidal effects of THD: THD induced autophagy by upregulating AMPK activity in the GBM cell lines [21]. To verify whether the THD analogs had a similar mechanism as that of THD in the GBM cells, the protein level in the THD-analog-treated GBM cells was analyzed using Western blotting. The data revealed that both THD analogs significantly increased the LC3-II and phospho-AMPK (Thr172) expression levels in a dose-dependent manner (Figure 1D). This result indicated that the THD analogs and THD may share the same biological mechanism in regulating AMPK activity.

We determined the cytotoxicity and effect of THD on the proliferation of GBM cell lines (U87MG and GBM8401). As shown in Figure 1E, THD significantly inhibited cell viability in a dose-dependent manner. Cell death was significantly increased after 24 h of treatment with 5, 10, and 15 µM THD, as assessed using the cell count method. Furthermore, THD (15 µM) markedly reduced the cell viability of the U87MG and GBM8401 cells in a time-dependent manner compared with that of the untreated cells (Figure 1F). Thus, all subsequent experiments were performed using 0, 5, 10, and 15 µM THD.

### 2.2. THD Induced Cell Cycle Arrest and Apoptosis in GBM Cells

**Figure 2:**
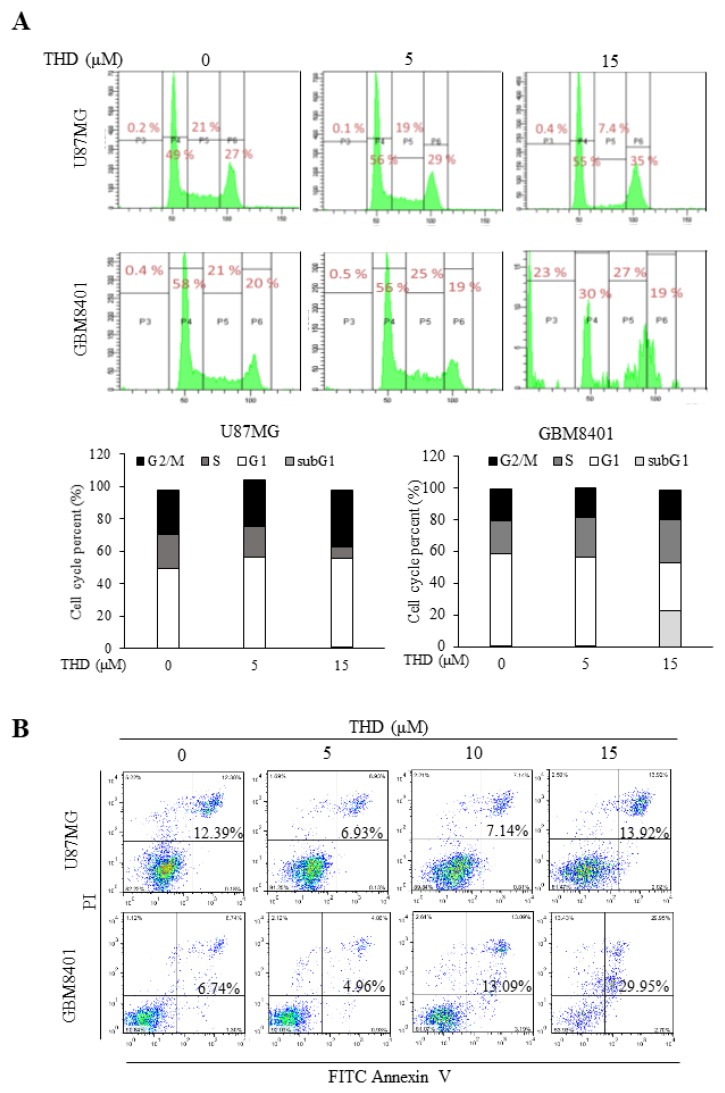
THD induced cell apoptosis in GBM cells. (**A**) U87MG and GBM8401 cells were treated with THD at 5 or 15 µM for 24 h, and cell cycle alterations were quantified through flow cytometry with staining with 50 µg/mL PI; (**B**) U87MG and GBM8401 cells were treated with THD at 5, 10, or 15 µM for 24 h, and the apoptotic cell percentage was quantified through flow cytometry by using the FITC Annexin V Apoptosis Detection Kit (BD PharmingenTM, cat. 556547). Representative scatter plots of PI (y-axis) versus annexin V (x-axis).

To evaluate the possible mechanisms through which THD inhibited cell growth, cell cycle profiles were assayed using flow cytometry. As illustrated in Figure 2A, the cell cycle profile of the GBM8401 cells was G1 58%, S 21%, G2/M 20%, and Sub G1 0.4%, and that of the U87MG cells was G1 49%, S 21%, G2/M 27%, and Sub G1 0.2%. Treatment with 5 µM THD did not alter the cell cycle profile. After treatment with 15 µM THD, the cell cycle profile of the U87MG cells was G1 55%, S 7.4%, G2/M 35%, and Sub G1 0.4%, and that of the GBM8401 cells was G1 30%, S 27%, G2/M 19%, and Sub G1 23%. Treatment with 15 µM THD for 24 h increased the G1 phase to 55% in the U87MG cells and the Sub G1 phase to 23% in the GBM8401 cells (Figure 2A). Thus, THD significantly increased the number of cancer cells in the G1 and Sub G1 phases, indicating THD-induced cell cycle arrest (U87MG) and cell death (GBM 8401). 

To elucidate whether the THD-induced inhibition of cell growth was associated with cell apoptosis induction, we investigated the effects of THD on apoptosis in the GBM cell lines. The apoptotic effects of THD were evaluated using flow cytometry after the cells were stained with annexin V and PI. Annexin V could be detected in both the early and late stages of apoptosis, whereas PI could be detected only in the late apoptosis or necrosis stage. Early apoptotic cells were positive for annexin V and negative for PI (lower right quadrant); late apoptotic or necrotic cells stained for both annexin V and PI (upper right quadrant). The data in Figure 2B show that incubation with 0–15 µM THD for 24 h increased the number of late apoptotic cells in GBM4801, and correlated with the subG1 result. These results demonstrate that THD can significantly induce cell apoptosis.

### 2.3. Effects of THD-Induced Apoptosis and Autophagy Were Mediated by P62 But Not P53 or Beclin-1

**Figure 3:**
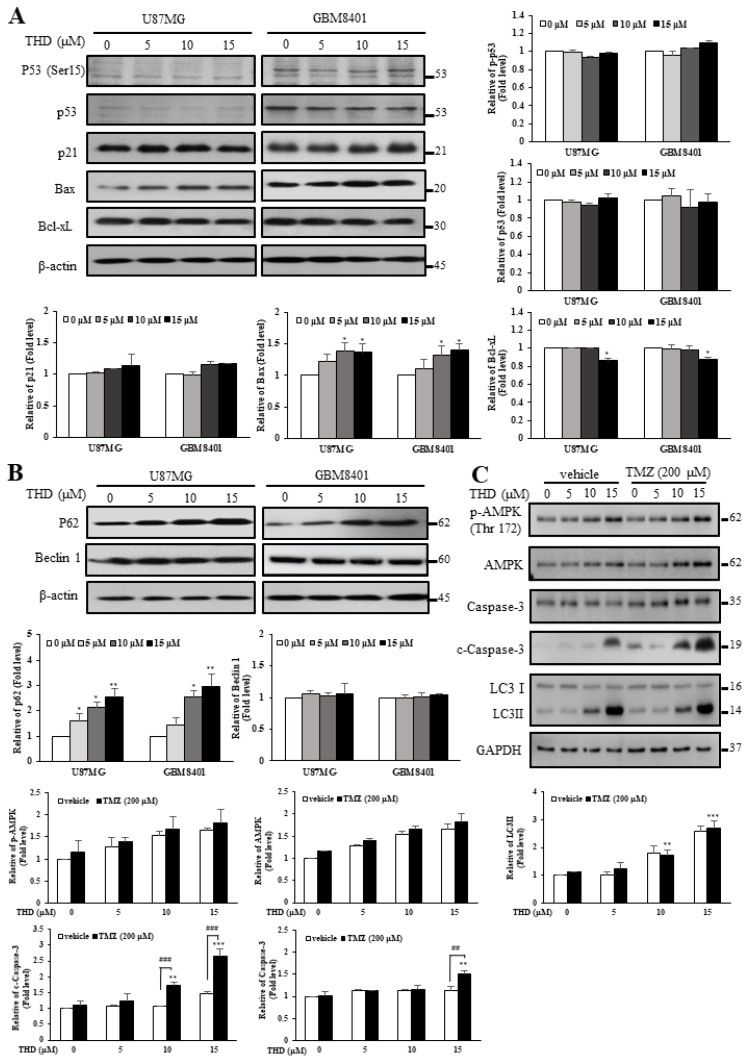
THD induced autophagy and apoptosis in GBM cells. (**A**) Cells were treated with indicated concentrations of THD for 24 h and subjected to Western blotting with antibodies against Bax, Bcl-xL, P53, P53 (Ser15), and p21; (**B**) Cells were treated with THD for 24 h, and Western blotting was performed to evaluate autophagy-related proteins, P62 and beclin-1; (**C**) Combination treatment with TMZ and THD induced AMPK activation and autophagy in GBM cells. U87MG cells were treated with 200 µM TMZ combined with different doses of THD for 24 h. p-AMPK (Thr172), AMPK, caspase-3, c-caspase-3, LC3-I, and LC3-II were detected using Western blotting. β-actin and GAPDH were used as an internal control. Bar graph represents mean of triplicates ± SD. * *p* < 0.05, ** *p* < 0.01, *** *p* < 0.001 compared with the control group. ^##^ *p* < 0.01, ^###^ *p* < 0.001 indicated TMZ (200 µM) vs. Vehicle.

Because sustained cell cycle arrest and senescence were believed to be mediated through P53-dependent upregulation of p21 [22], we next examined P53, phosphorylated P53 (ser15), and p21 protein levels in the cells. Western blotting confirmed the ineffectiveness of THD in both U87MG (p53-Wiletype) and GBM8401 (p53-mutated) cells (Figure 3A). These results suggested that the growth inhibitory properties of THD may be caused through P53-independent pathways. The Bcl-2 protein family determines the commitment of cells to apoptosis. The antiapoptotic members of this family, such as Bcl-2 and Bcl-xL, prevent apoptosis either by sequestering preforms of death-driving cysteine proteases or by preventing the release of mitochondrial apoptogenic factors into the cytoplasm. We further evaluated the involvement of the Bcl-xL protein family proteins through Western blotting. As presented in Figure 3A, THD caused a marked decrease in Bcl-xL expression but engendered a significant increase in Bax expression.

In addition to apoptosis, autophagy plays crucial roles in cancer cell survival and death; thus, it is gaining increasing interest in cancer research. Beclin-1 interacts with several binding partners and can both induce and suppress autophagy pathways [23]. While numerous commonly used anticancer drugs have been reported to induce apoptosis in cancer cells, autophagy was also frequently observed in response to these drugs, including THD, TMZ, ABT-737, and sorafenib [3,4,21,24,25]. In our previous study, we found that THD and its analogs might enhance LC3-II-induced autophagy in GBM cells (Figure 1D). In the current study, we investigated whether THD promotes autophagy in a Beclin-1- and P62-dependent manner. After treatment of the U87MG and GBM8401 cells with different concentrations (0–15 µM) of THD for 24 h, the expression level of P62 significantly increased in a dose-dependent manner, but that of Beclin-1 did not (Figure 3B). Altogether, these data suggest that THD-mediated autophagy could occur through P62, but not P53 or Beclin-1.

TMZ is a frequently used chemotherapeutic agent for GBM treatment. A study reported that AMPK activation has potential effects on TMZ-induced GBM cell death [26]. In addition, AMPK activation was involved in THD-induced autophagy [21]. Studies have revealed that treatment with ABT-737 (a BH3 mimetic agent) combined with TMZ exerted a superior effect on apoptosis induction in U87MG cells, which effectively reactivated apoptotic markers [3,4]. Thus, to investigate the effect of a combination of THD and TMZ, U87MG cells were treated with different doses of THD combined with 200 µM TMZ for 24 h. As illustrated in Figure 3C, the level of phospho-AMPK (Thr172) was upregulated in the U87MG cells after the combination treatment. In addition, administering TMZ and THD induced autophagy and upregulated the level of LC3-II in a dose-dependent manner (Figure 3C). These results suggested that excessive autophagy through AMPK activation plays a critical role in the mechanism of this drug combination. Taken together, these results further suggested that the mechanism of THD-induced cytotoxicity is mainly through unbalanced autophagy, leading to apoptosis in GBM cells.

### 2.4. Pathway Analysis of Differentially Expressed Gene Signatures through Microarray Profiling

The original target of THD in patients with psychosis was the dopamine receptor, which is rarely found in GBM cell lines, according to our previous data [21]. To identify the target of THD in the GBM cell lines, GBM8401 cells were treated with 10 µM THD for 24 h and then subjected to microarray profiling.

**Figure 4:**
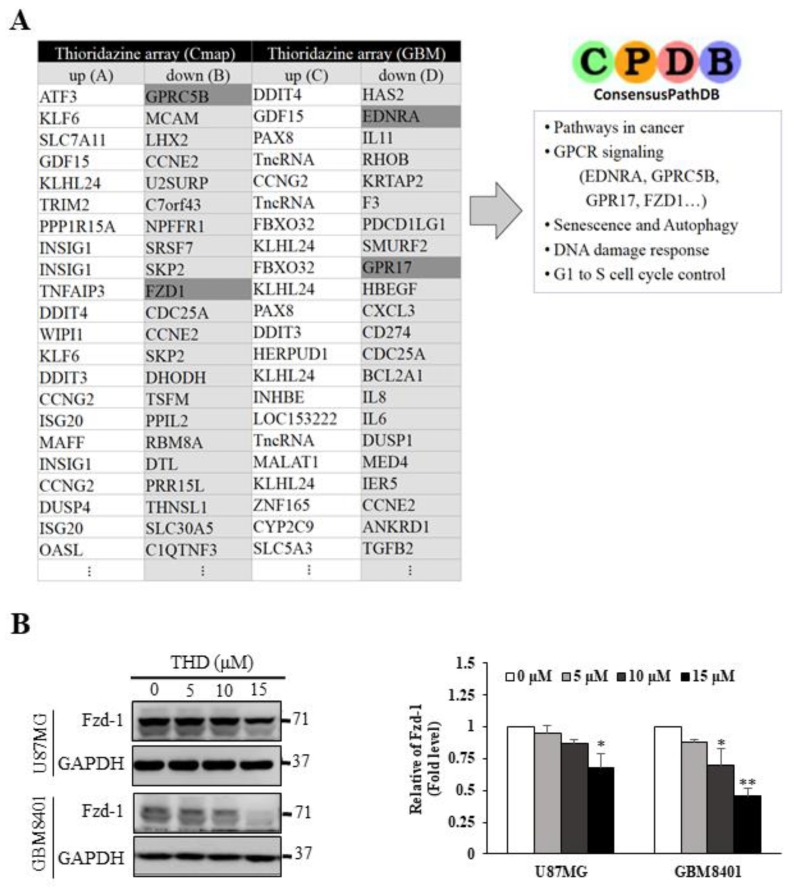
GPCRs targets of THD. (**A**) Two databases were used to identify possible pathways involved in THD-induced autophagy and apoptosis by using the ConsensusPathDB pathway analyzer. The most significant pathways include cancer-related pathways, G-protein-coupled protein signaling, senescence and autophagy, DNA damage response, and G1 to S cell cycle control; (**B**) Candidate protein Fzd1 was validated on GBM8401 and U87MG with 24-h treatment with 5, 10, or 15 µM THD. GAPDH was used as an internal control. Bar graphs represent the mean of triplicates ± SD. * *p* < 0.05, ** *p* < 0.01, compared with the control group.

Using the ConsensusPathDB database (http://cpdb.molgen.mpg.de/), we performed a pathway analysis of the differentially expressed gene signatures generated from the microarray profiling process. Several THD-mediated pathways were highlighted, including cancer, G-protein-coupled receptor (GPCR) signaling, senescence and autophagy, DNA damage response, and G1 to S cell cycle control pathways (Figure 4A). Notably, GPCRs are membrane-associated proteins and have been widely recognized as drug targets [27,28]. By using micro-Western blotting to validate these candidate proteins in a patient with GBM, we confirmed the upregulation of p-AMPKα (Thr 172) and downregulation of p-mTOR (Ser 2481) in a dose-dependent manner (Table S1). Thus, we evaluated whether GPCRs may be the targets of THD. The GBM8401 and U87MG cells were treated with 5, 10, or 15 µM THD for 24 h, and the lysates were subjected to Western blot analysis. As shown in Figure 4B, one of the GPCRs, Fzd, was downregulated in the cells after treatment with THD, suggesting that THD-elicited pathways may be modulated through Fzd. Notably, gene expression of several GPCRs, particularly for the Fzd1 level, was consistent with the expression observed in five cohorts of patients with GBM from 2.0 to 4.0 (Figure S1).

### 2.5. THD Mediated β-Catenin Degradation through the Wnt/β-Catenin Signaling Pathway

**Figure 5:**
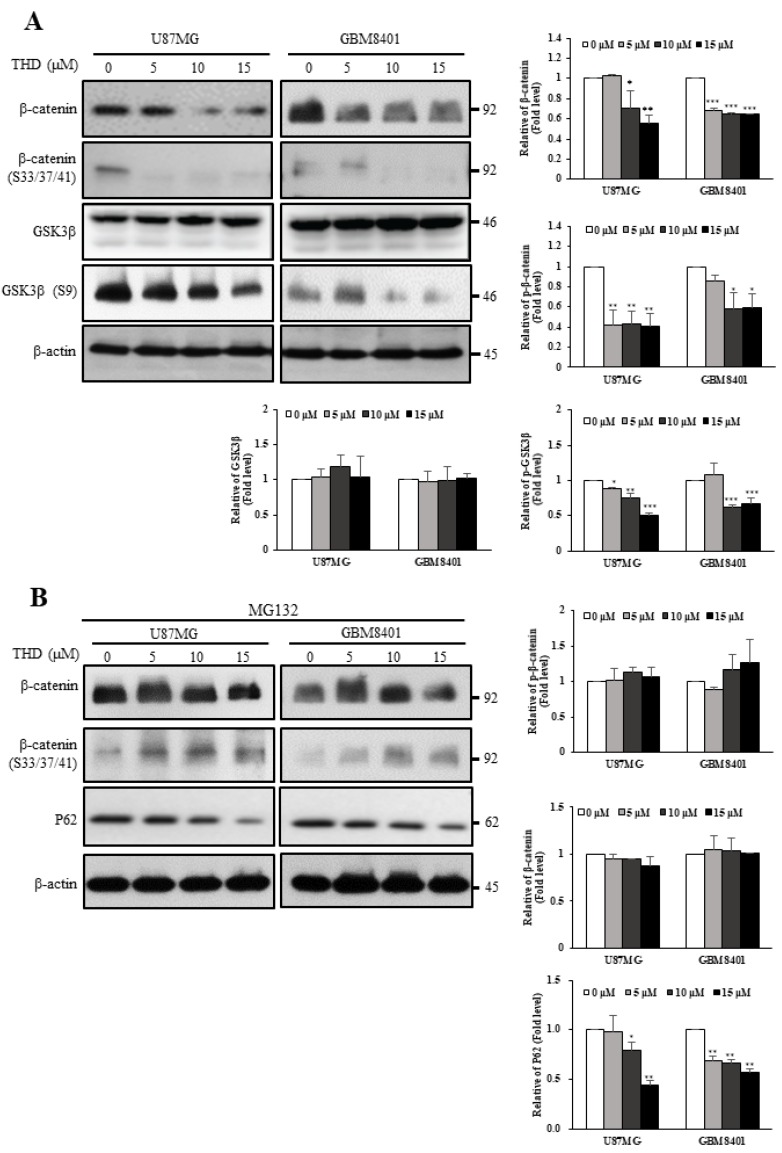
THD mediated β-catenin degradation through Wnt/β-catenin signaling pathway. (**A**) GBM cells were treated with 5, 10, or 15 µM THD for 24 h, and the protein expression patterns of the Wnt pathway were determined; (**B**) GBM cells were incubated with different concentrations of THD and MG-132 (10 µM) for 24 h. Cytosolic fractions were prepared and subjected to Western blotting with GSK3β, phosphor S9 GSK3β, β-catenin, phosphor S33/37/41-β-catenin and P62 antibodies. β-actin was used as an internal control. Bar graphs represent the mean of triplicates ± SD. * *p* < 0.05, ** *p* < 0.01, *** *p* < 0.001 compared with the control group.

Fzd is upstream of the Wnt signaling pathway and regulates the relative stability of β-catenin through GSK-3β-dependent phosphorylation [29,30]. As presented in Figure 5A, THD reduced the protein levels of β-catenin and phospho-β-catenin (Ser33/Ser37/Thr41), phosphorylated by GSK3β in the U87MG and GBM8401 cells. In addition, the phosphorylation level of GSK3β Ser9 decreased with THD treatment, indicating that the GSK3β activity increased and phosphorylated β-catenin [31]. To verify that the decreased protein level of β-catenin was caused by protein degradation, the proteasome inhibitor MG132 was applied to the THD-treated cells. As expected, THD-induced β-catenin degradation was reversed and P62 expression was also suppressed when MG132 was applied (Figure 5B). These results clearly demonstrated that THD enhanced GSK3β activity through Ser9 phosphorylation downregulation, which in turn enhanced β-catenin phosphorylation at Ser33/Ser37/Thr41, which triggered protein degradation. We concluded that THD indeed mediated β-catenin degradation through the Wnt/β-catenin signaling pathway.

## 3. Discussion

THD has been reported to exert anticancer effects by inhibiting proliferation, inducing apoptosis, countering metastasis, and reversing multidrug resistance; the anticancer ability might result from apoptosis through autophagy regulation [16,21]. According to our results, THD suppressed GBM cell line growth and induced sub-G1 accumulation and late apoptosis in a dose-dependent manner (Figure 2A,B). THD treatment activated the proapoptotic Bcl-2 family member Bax and reduced the expression of the antiapoptotic protein Bcl-xL (Figure 3A). Of note, the p53 tumor suppressor is induced by various stress stimuli and coordinates an adaptive gene expression program, leading to growth arrest or cell death [32]. In the present study, the THD-induced apoptosis in the p53-mutated GBM8401 and p53-Wildtype U87MG cell line was associated with increased Bax and decreased Bcl-xL levels, indicating that cell death may result from Bax translocation and caspase activation (Figure 3A). To our knowledge, this is the first article to describe THD-induced apoptosis through P53-independent pathways.

A notable finding of this study is that changes in the level of P62 seemed to respond more rapidly to and exhibit a greater correlation with the outcome of THD-induced cell death. Previous research revealed that P62 depletion could inhibit the recruitment of LC3 to autophagosomes under starvation conditions and that the background level of LC3-II was higher in cells overexpressing P62 than in other cells, suggesting that autophagic activity is greater when P62 levels are higher [33]. Additionally, P62 acts as a signaling core in orchestrating apoptosis [9]. In response to death receptor stimulation, Cullin3-based ubiquitin ligases can combine with death-inducing signaling complexes and induce the polyubiquitination of caspase-8 [34]. When cells were treated with reagents inducing ER stress or proteasome inhibition, the cells could activate the apoptosis system directly through caspase-8, without the involvement of death receptor signaling [35]. This novel mechanism of caspase-8-mediated apoptosis was dependent on the autophagy-related proteins LC3 and P62 [34,35,36]. Thus, in addition to serving as a typical caspase species in the “classic” extrinsic apoptosis, caspase-8 can be activated in a P62-dependent manner and can be involved in an alternative endogenous apoptotic pathway, particularly when induced by various reagents or drugs. Our study in 2015 demonstrated the activation of caspase-8 after THD treatment [21]. Thus, THD-induced effects in the GBM cells resulted from P62-activated apoptosis, accompanied by autophagy.

It is well-known that TMZ is a chemotherapeutic agent frequently used in GBM treatment [1,2,3,4,5,37]. However, the cytotoxic activity of TMZ was reported to be inversely dependent on the DNA repair enzyme O6-methylguanine DNA methyltransferase (MGMT) and to be dependent on the mismatch repair pathway [37]. Therefore, identifying novel therapeutic approaches for GBM is urgently required; however, discovering new drugs is difficult because of the blood–brain barrier (BBB). Several other compounds such as hydroxychloroquine and BAF A1 inhibit late-stage autophagy. Nevertheless, because of its high lipophilicity, THD accumulates in the brain, rendering it an ideal compound for targeting intracranial malignancies [38]. Our results demonstrate that THD, a commonly used drug for treating psychosis, reduces the cell viability of GBM cell lines. Moreover, THD can cross the BBB and is clinically approved; thus, the novel indication could be fast-tracked into clinical trials. In this study, the data showed that the therapeutic effect of the dose above 10 µM increased autophagy, and the THD-TMZ combination treatment induced apoptosis and autophagy, indicating that THD might still have therapeutic action against cancer by promoting autophagy (Figure 3C). From our basis, modification of THD to improve the IC_50_ shall provide a promising target for treating GBM.

**Figure 6:**
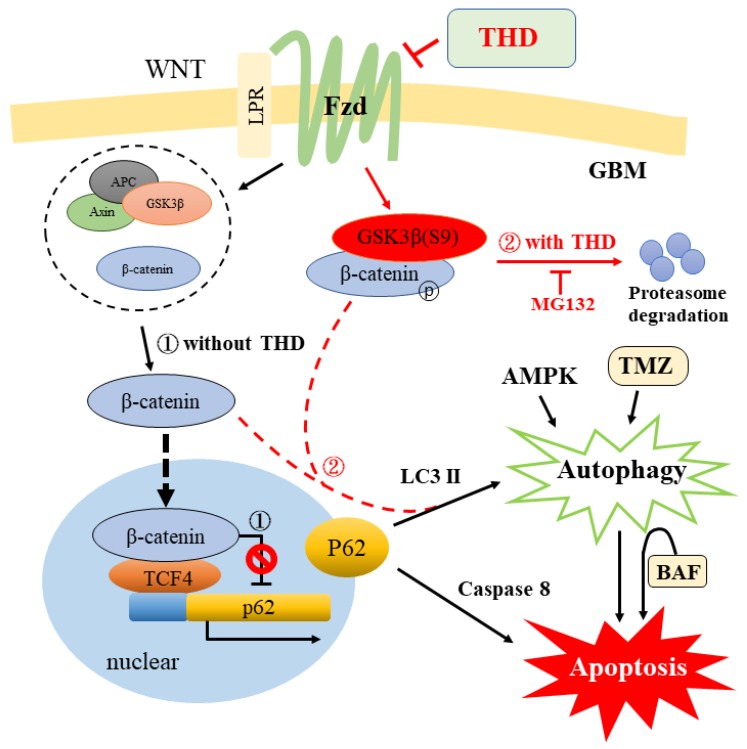
Model for THD on a crosstalk in Fzd/GSK3β/β-catenin/P62 axis. A working model summarizing the possible pathways obtained in various studies (References [10,21] and this study) revealed a regulatory feedback mechanism that places β-catenin-LC3 II/P62-caspase-8 in a loop of proliferation, autophagy, and apoptosis, with implications for THD antipsychotics targeting of these pathways for GBM cells to THD. Route 1, without THD; route 2, with THD; and dashed red lines indicate possible mechanisms (for details, see the Discussion Section 3.).

On the basis of the preceding observations, we suggest a Fzd/GSK3β/β-catenin/P62 axis working model, as shown in Figure 6. In the canonical Wnt/β-catenin signaling pathway, in the presence of Wnt, the destruction complex (comprising Axin, APC, GSK3β, and β-catenin; dashed circle) disintegrates and β-catenin is stabilized, subsequently binding to TCF in the nucleus to upregulate target genes for cell proliferation and repress P62 (route 1). Importantly, the Wnt signaling pathway is the stabilization of β-catenin, which is regulated by its phosphorylation and subsequent degradation [39]. In this study, we observed that triggering THD on the GBM cells reduced the expression of Fzd and GSK3β S9 phosphorylation, which indicates increased activity of GSK3β, resulting in an increase in phosphorylated β-catenin and its degradation (route 2). Notably, inhibiting both TCF-β-catenin and autophagy flux reduced cell viability and induced apoptosis through a P62-dependent mechanism involving caspase-8 (route 2; dashed red lines). Taken together, these results further suggest that TMZ and THD induced cytotoxicity mainly through excessive autophagy, leading to apoptosis in GBM cells. Our studies (Reference [21] and this study) have revealed a regulatory feedback mechanism that places β-catenin-LC3 II/P62-caspase-8 in a loop of proliferation, autophagy, and apoptosis to be compromised, with implications for drug targeting of these pathways for GBM cells to THD [21]. In conclusion, THD induces apoptosis in GBM cells through attenuated Wnt/β-catenin signaling, leading to p62-mediated autophagy with caspase-8 activation. Importantly, the combined THD and TMZ exert a better synergistic effect in inducing apoptosis that could serve as an alternative therapeutic drug for repositioning in GBM.

## 4. Materials and Methods

### 4.1. Cell Culture and Chemicals

The human glioblastoma U87MG (Cat# HTB-14, p53-WT, MGMT negative) cell line was obtained from the American Type Culture Collection (Manassas, VA, USA). GBM 8401 (Cat# 60163, p53-mutated, MGMT negative) was obtained from the Bioresource Collection and Research Center (Hsinchu, Taiwan). Cells were cultured in minimum essential medium (Gibco; Thermo Fischer Scientific, Grand Island, NY, USA) and RPMI-1640 (Gibco) supplemented with 10% fetal bovine serum (Gibco), 2 mM L-glutamine (Gibco), and antibiotics (penicillin/streptomycin, 100 IU/L; Gibco). THD (Sigma, Aldrich, St. Louis, MO, USA) and its analogs were prepared in our lab.

### 4.2. Cell Viability Assay

Cell viability was measured using the sulforhodamine B (SRB, Abcam, ab235935) and Cell Counting Kit-8 (CCK-8, sigma 96992, St. Louis, MO, USA) assays based on the measurement of cellular protein content. U87MG and GBM8401 cells were seeded (2000 cells/well) in a 96-well plate and then treated with various concentrations of THD for 24 h. Subsequently, the cells were fixed with 100 mg/mL trichloroacetic acid and stained with 57 µg/mL SRB solution for 1 h and then washed repeatedly with 10 mg/mL acetic acid. The protein-bound dye was dissolved in 10 mM Tris base solution to obtain a reading at 540 nm. The cell viability of each treated sample was measured using the CCK-8 kit according to the manufacturer’s instructions. The absorbency of cells was measured using a 96-well plate reader at 450 nm.

### 4.3. Apoptosis Analysis

The Annexin V-fluorescein isothiocyanate (FITC)-binding assay was used to quantify the percentage of early and late apoptosis. Cells (2 × 10^5^ cells/well) were seeded in a 24-well plate and incubated for 24 h; they were then treated with the indicated THD concentration for another 24 h. Both adherent and nonadherent cells were harvested prior to staining with Annexin V-FITC and propidium iodide (PI) according to the manufacturer’s instructions. 

### 4.4. Cell Cycle Analysis

Cell cycle quantification was performed to determine the alteration in each cell phase. Cells (2 × 10^5^ cells/well) were seeded in a 24-well plate and incubated for 24 h; they were then treated with the indicated THD concentration for another 24 h. The cells were harvested and re-suspended in 70% ethanol in phosphate buffered saline (PBS) and incubated at −20 °C for 1 h. Subsequently, the cells were re-suspended in cold PBS containing 20 µg/mL PI and 100 µg/mL RNAse A (Sigma). The cells were then incubated in a dark chamber for 30 min at room temperature, after which the DNA content was analyzed through BD FACScan using FACS Diva software (BD Biosciences, San Jose, CA).

### 4.5. Western Blot Analysis

Cells were lysed with immunoprecipitation assay lysis buffer (Protech Inc., Taipei, Taiwan), and the protein concentration was quantified using a protein assay reagent (Bio-Rad Laboratories, Inc., Hercules, CA, USA). Each lane containing 20 µg of total protein was subjected to sodium dodecyl sulfate-polyacrylamide gel electrophoresis and then transferred to a polyvinylidene difluoride membrane. The following primary antibodies were used: phospho-AMPK (Thr-172; 1:1000; 2535S), AMPK (1:1000; 2532S), c-caspase-3 (1:1000; 9664S), caspase-3 (1:1000; 9662S), Beclin1 (1:1000; 3495S), Bax (1:1000; 2772S), p21(1:1000; 2947S), Bcl-xL (1:1000; 2764S), and phospho-P53 (p15; 1:1000; 9284S), obtained from Cell Signaling. P62 (1:2000; ab56416) and β-actin (1:1000; ab3280) were obtained from Abcam (Cambridge Science Park, UK); LC3 was obtained from Abgent (San Diego, CA, USA; 1:1000; AP1802a); and P53 (1:1000; GTX70216), Fzd 1 (1:1000; GTX108181), GAPDH (1:10,000; GTX100283), and α-tubulin (1:5000; GTX112141) were obtained from GeneTex (Irvine, CA, USA). After protein hybridized with the primary antibody followed with secondary horseradish peroxidase-conjugated antirabbit and antimouse antibodies (Chemicon, Shinagawa-ku, Tokyo, Japan; 12-348 and 12-349, respectively), protein was visualized using an enhanced chemiluminescence reagent (GE Healthcare, Pittsburgh, PA, USA; RPN2108) on an X-ray film. 

### 4.6. Clonogenic Assay

The GBM8401 cells were seeded at a density of 1000 cells/well in 6-well plates. After 10 days, the cells were washed twice with PBS, fixed with a solution mixture of acetic acid and methanol (1:3), and then stained with 0.5% crystal violet in methanol at room temperature. The colonies were carefully washed with tap water, and then colonies with a diameter larger than 50 µM were counted under a microscope. 

### 4.7. Microarray Analysis

After 6 h of treatment with 10 µM THD, total RNA was extracted from the GBM8401 cells by using an RNeasy Mini kit (QIAGEN, Valencia, CA, USA; 74106). The quality of total RNA was determined using an ultraviolet spectrophotometer and Agilent 2100 Bioanalyzer and had an OD260/OD280 ratio ranging from 1.9 to 2.1. Total RNA was used to synthesize cDNA, followed by labeling, fragmentation, and quality check using the bioanalyzer. According to instructions provided by Affymetrix (http://www.affymetrix.com/support/technical/manuals.affx), hybridization, washing, and staining procedures were performed. Images containing intensity information were converted into text files by using GeneChip Operating Software, developed by Affymetrix. The microarray data sets were determined using GeneSpring 7.31 software (QIAGEN, Agilent, Santa Clara, CA, USA).

### 4.8. Consensus PathDB Data Analysis

Two data sets were used for THD-elicited pathway analysis, namely the Cmap database, which contains 14 THD-treated Affymetrix U133A arrays without GBM cells, and our Affymetrix HG-U133 Plus 2 microarray database generated from THD-treated GBM 8401 cells. For the Cmap database, genes were selected based on at least a two fold expression change and appearance in at least 3 of the 14 arrays. For the THD-treated GBM 8401 database, genes were selected based on at least a 1.75-fold expression change. Subsequently, genes that fitted the criteria were subjected to ConsensusPathDB analysis to identify THD-involving pathways. 

### 4.9. Statistical Analysis

Data are presented as mean ± standard deviation. Statistical analyses were performed using one-way analysis of variance. Data were compared using Student’s *t*-test. The level of statistical significance was set at *p* < 0.05.

## Supplementary Materials

Supplementary materials can be found at http://www.mdpi.com/1422-0067/20/3/473/s1. Figure S1. Gene expression of EDNRA and Fzd-1 in five cohorts of patients with GBM. Table S1. Upregulated and downregulated proteins in micro-Western assay.
