# Supplementary material for: Thioridazine Enhances P62-Mediated Autophagy and Apoptosis Through Wnt/β-Catenin Signaling Pathway in Glioma Cells
Source: Int J Mol Sci. 2019 Jan 22;20(3):473. doi: 10.3390/ijms20030473 (PMC6386927; doi:10.3390/ijms20030473)

Fig 4

A

Thioridazine array (Cmap)		Thioridazine array (GBM)	
up (A)	down (B)	up (C)	down (D)
ATF3	GPRC5B	DDIT4	HAS2
KLF6	MCAM	GDF15	EDNRA
SLC7A11	LHX2	PAX8	IL11
GDF15	CCNE2	TncRNA	RHOB
KLHL24	U2SURP	CCNG2	KRTAP2
TRIM2	C7orf43	TncRNA	F3
PPP1R15A	NPFFR1	FBXO32	PDCD1LG1
INSIG1	SRSF7	KLHL24	SMURF2
INSIG1	SKP2	FBXO32	GPR17
TNFAIP3	FZD1	KLHL24	HBEGF
DDIT4	CDC25A	PAX8	CXCL3
WIPI1	CCNE2	DDIT3	CD274
KLF6	SKP2	HERPUD1	CDC25A
DDIT3	DHODH	KLHL24	BCL2A1
CCNG2	TSFM	INHBE	IL8
ISG20	PPIL2	LOC153222	IL6
MAFF	RBM8A	TncRNA	DUSP1
INSIG1	DTL	MALAT1	MED4
CCNG2	PRR15L	KLHL24	IER5
DUSP4	THNSL1	ZNF165	CCNE2
ISG20	SLC30A5	CYP2C9	ANKRD1
OASL	C1QTNF3	SLC5A3	TGFB2
⋮	⋮	⋮	⋮

CPDB
ConsensusPathDB

- Pathways in cancer
- GPCR signaling
  (EDNRA, GPRC5B, GPR17, FZD1…)
- Senescence and Autophagy
- DNA damage response
- G1 to S cell cycle control

B

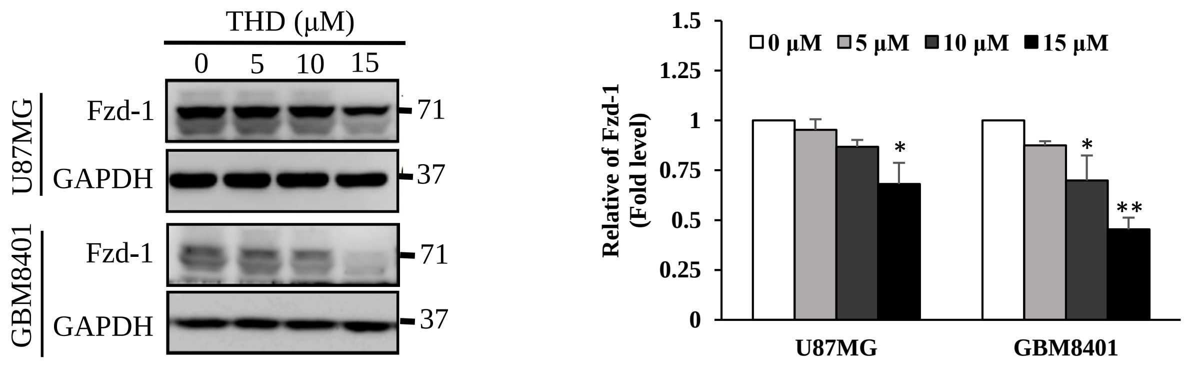

Supplement: Supplementary file 1 [file ijms-20-00473-s001.zip › PDF/Figure 4 Big.pdf]
