# Supplementary material for: Thioridazine Enhances P62-Mediated Autophagy and Apoptosis Through Wnt/β-Catenin Signaling Pathway in Glioma Cells
Source: Int J Mol Sci. 2019 Jan 22;20(3):473. doi: 10.3390/ijms20030473 (PMC6386927; doi:10.3390/ijms20030473)
Supplement: Supplementary file 1 [file ijms-20-00473-s001.zip › PDF/SUP.pdf]

Supplement Fig 1

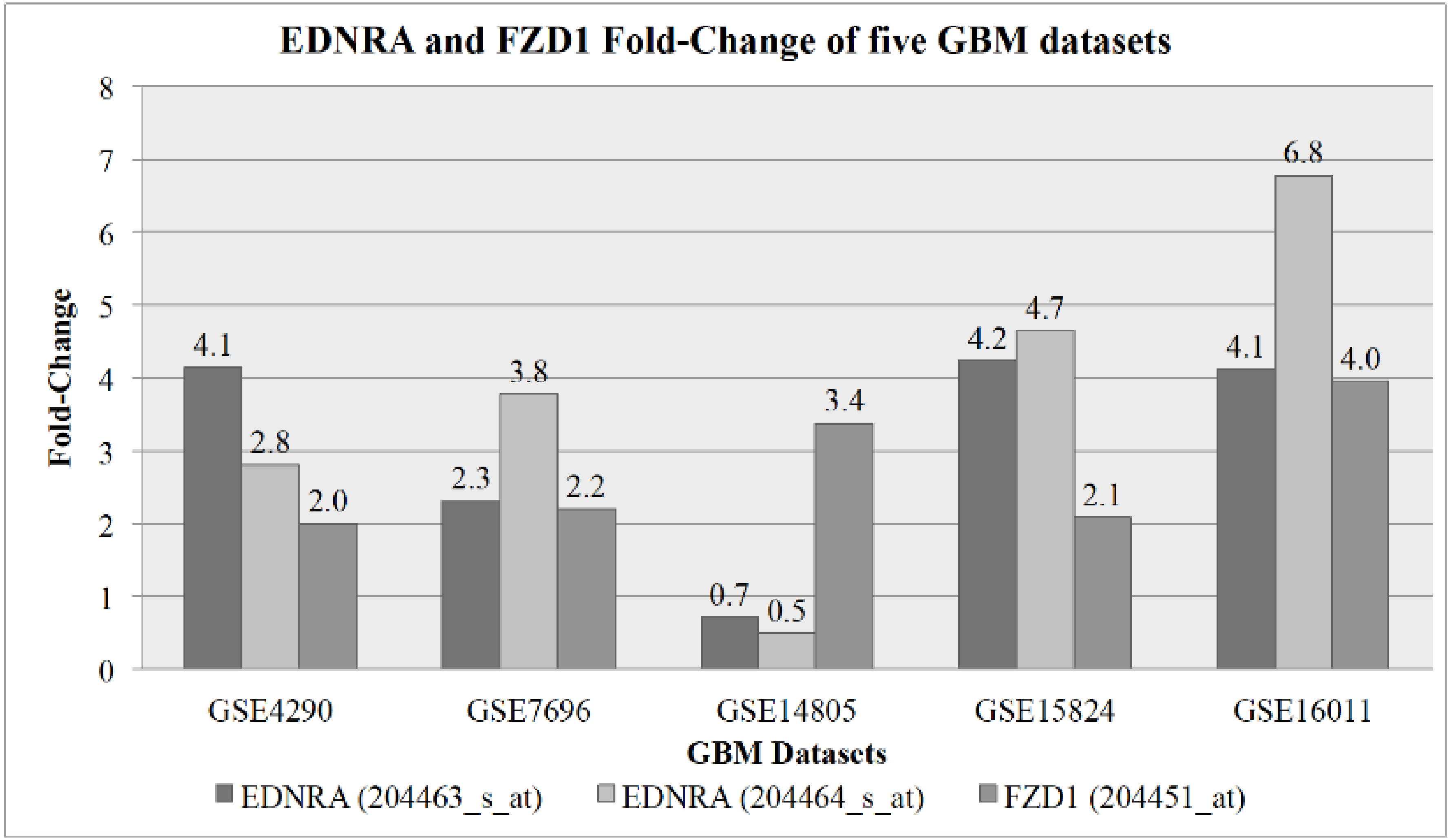

Supplementary table 1. Up-regulated and down-regulated proteins in the microwestern assay.

Up-regulation Protein	Sample	Fold change
p-AMPKα (Thr 172)	THD (0 μM)	1.000
	THD (5 μM)	1.287
	THD (10 μM)	1.620
	THD (15 μM)	3.403

Down-regulation Protein	Sample	Fold change
p-mTOR (Ser 2481)	THD (0 μM)	1.000
	THD (5 μM)	0.850
	THD (10 μM)	0.409
	THD (15 μM)	0.576
