# Supplementary figures and images for: Thioridazine Enhances P62-Mediated Autophagy and Apoptosis Through Wnt/β-Catenin Signaling Pathway in Glioma Cells
Source: Int J Mol Sci. 2019 Jan 22;20(3):473. doi: 10.3390/ijms20030473 (PMC6386927; doi:10.3390/ijms20030473)

**Fig 1**

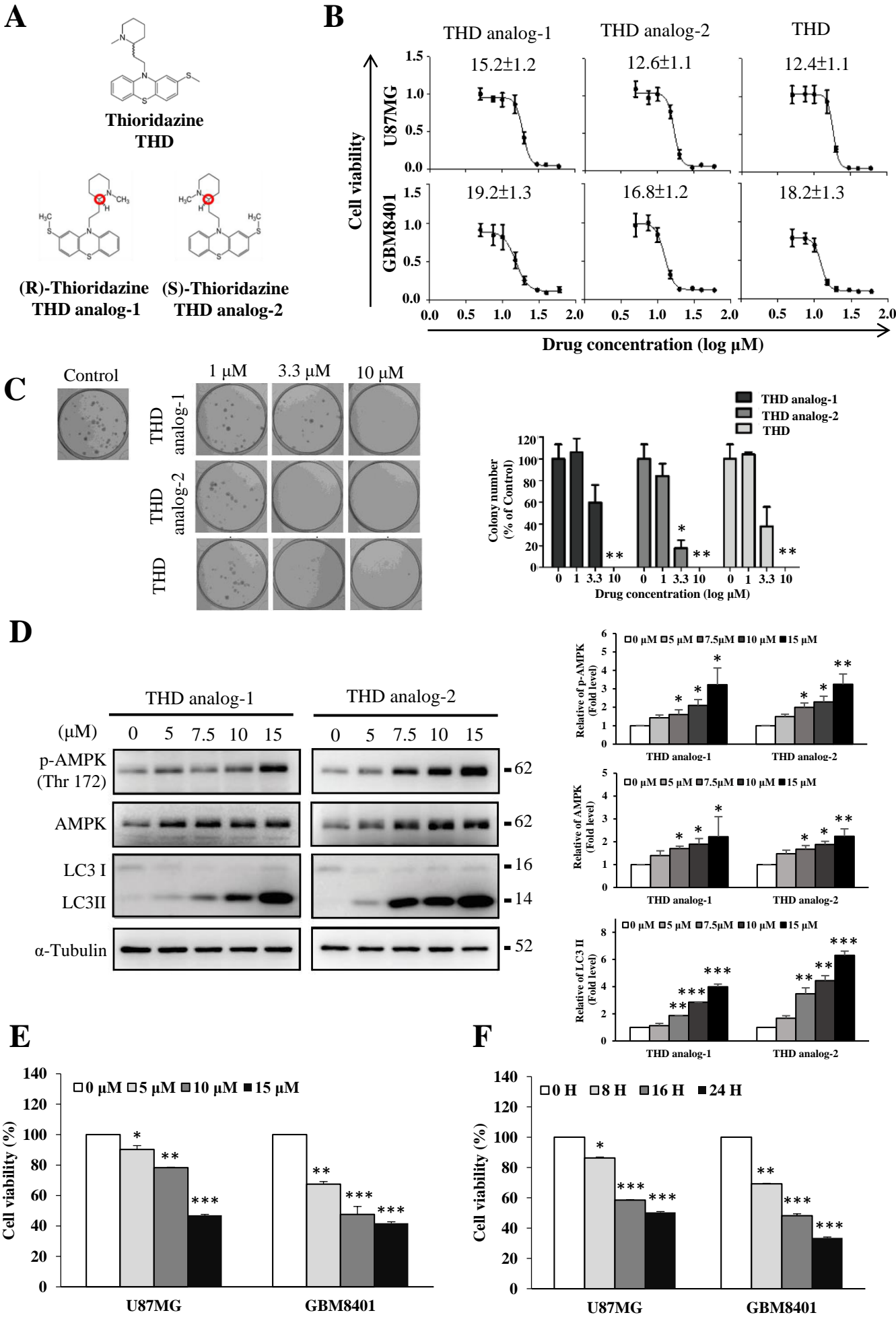

Supplement: Supplementary file 1 [file ijms-20-00473-s001.zip › PDF/Figure 1 Big.pdf]

# A

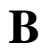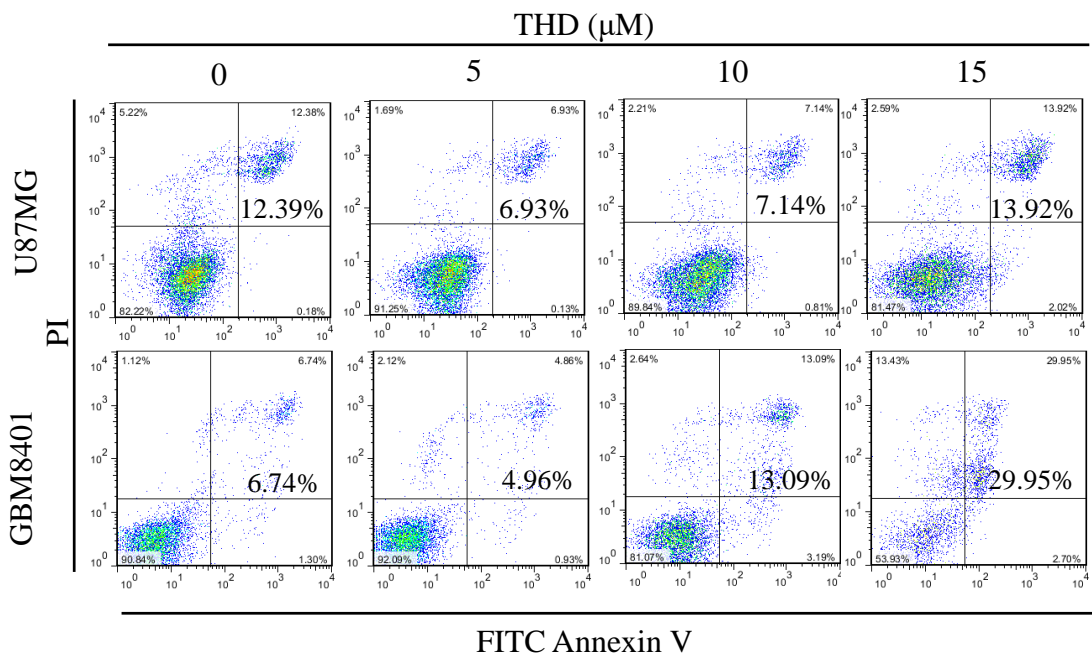

Supplement: Supplementary file 1 [file ijms-20-00473-s001.zip › PDF/Figure 2 Big.pdf]

# Fig 3

**A**

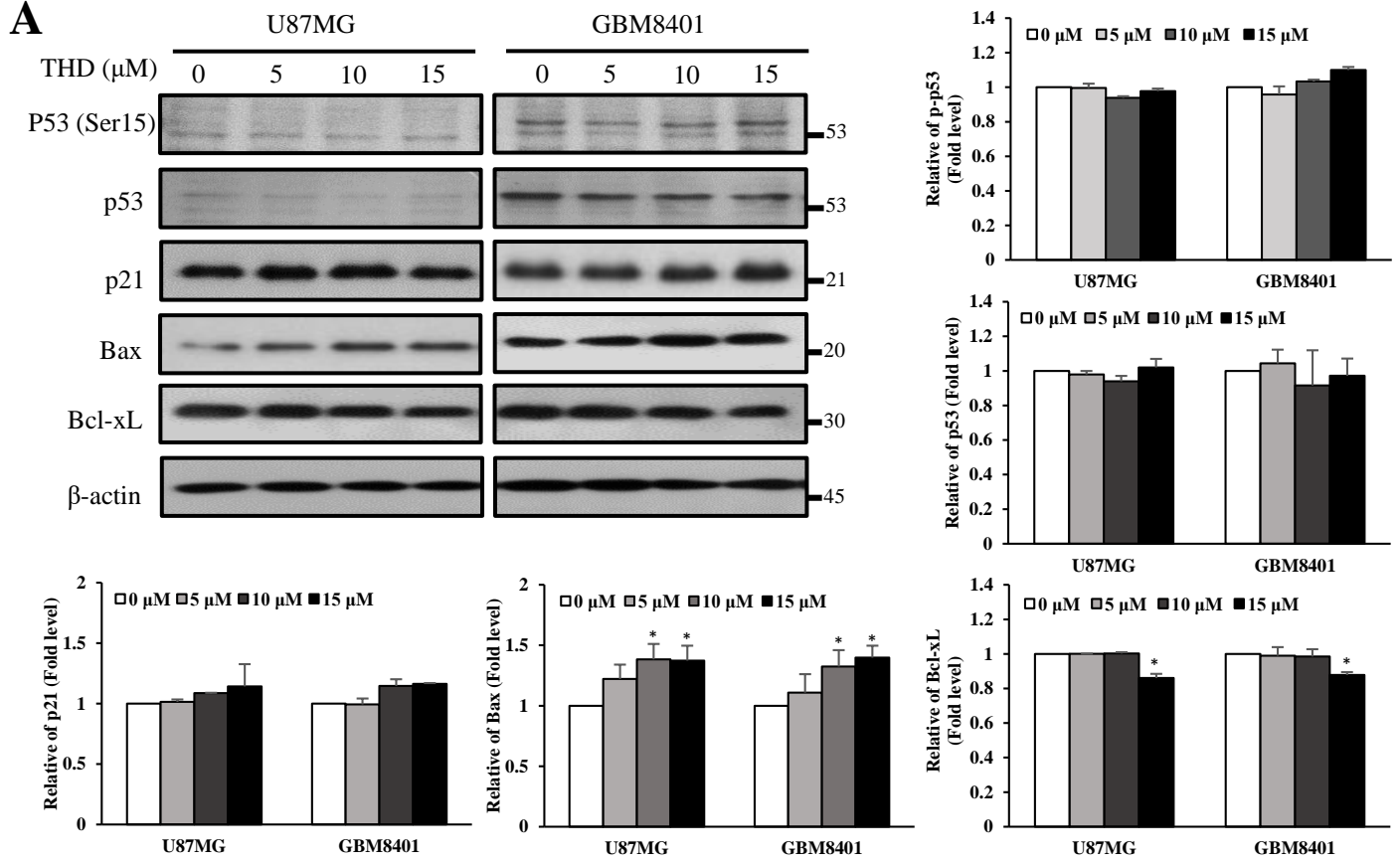

**B**

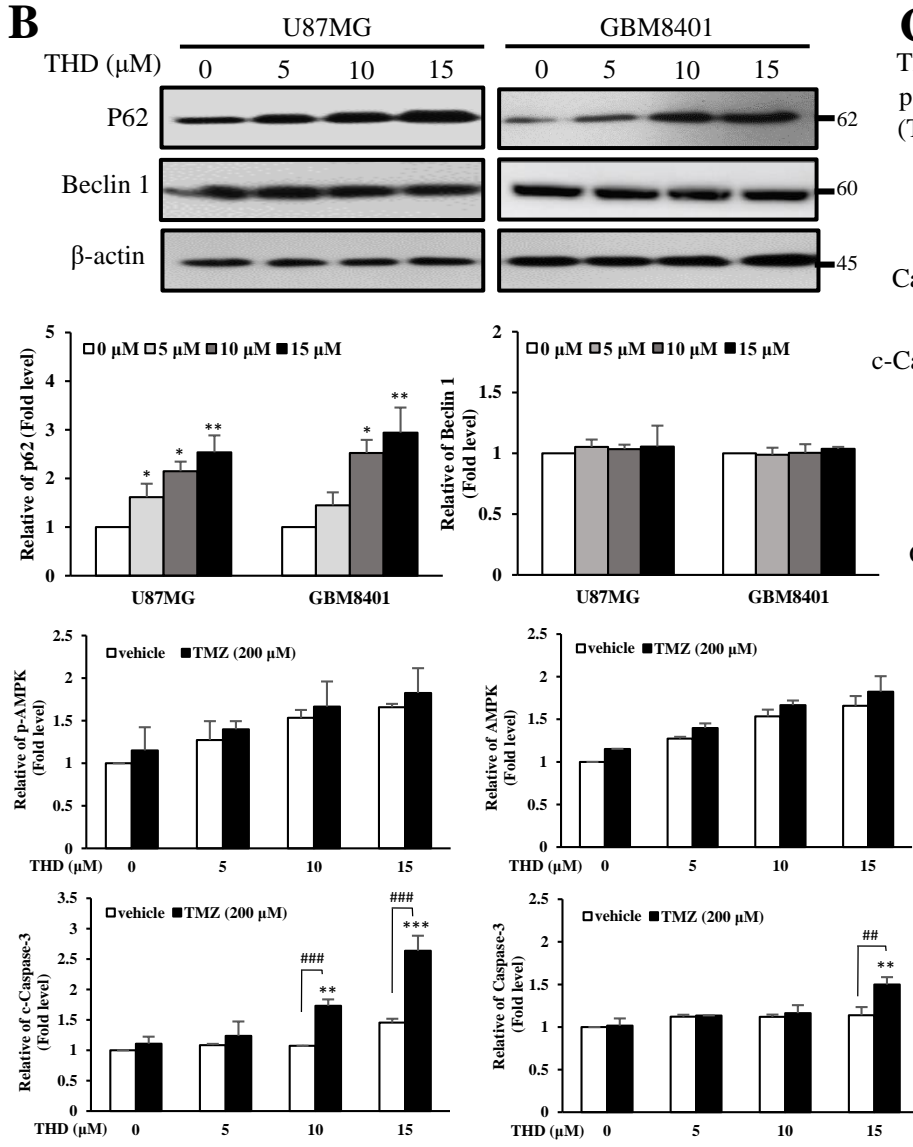

**C**

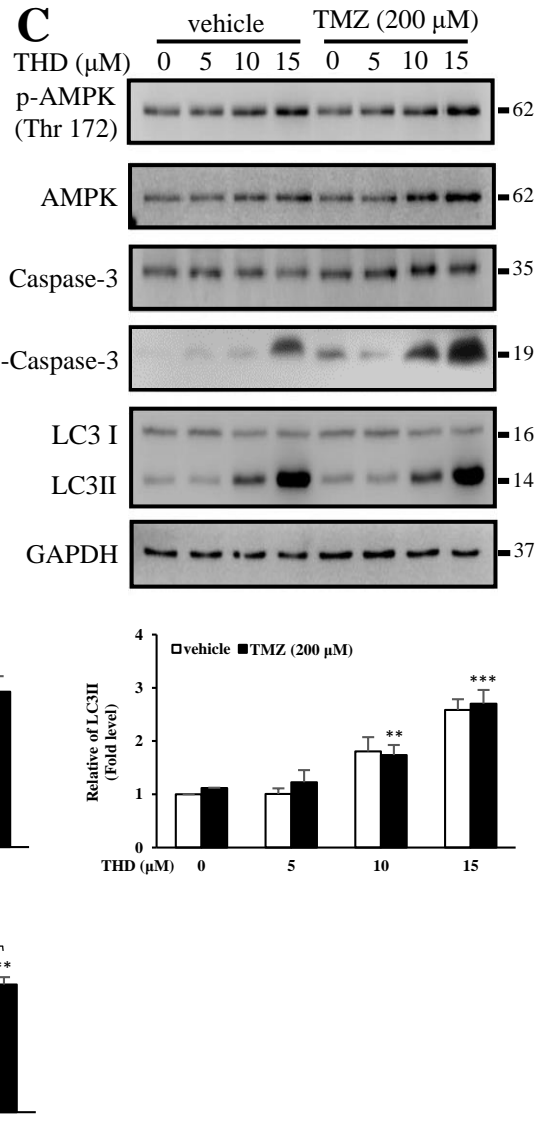

Supplement: Supplementary file 1 [file ijms-20-00473-s001.zip › PDF/Figure 3 Big.pdf]

Fig 5

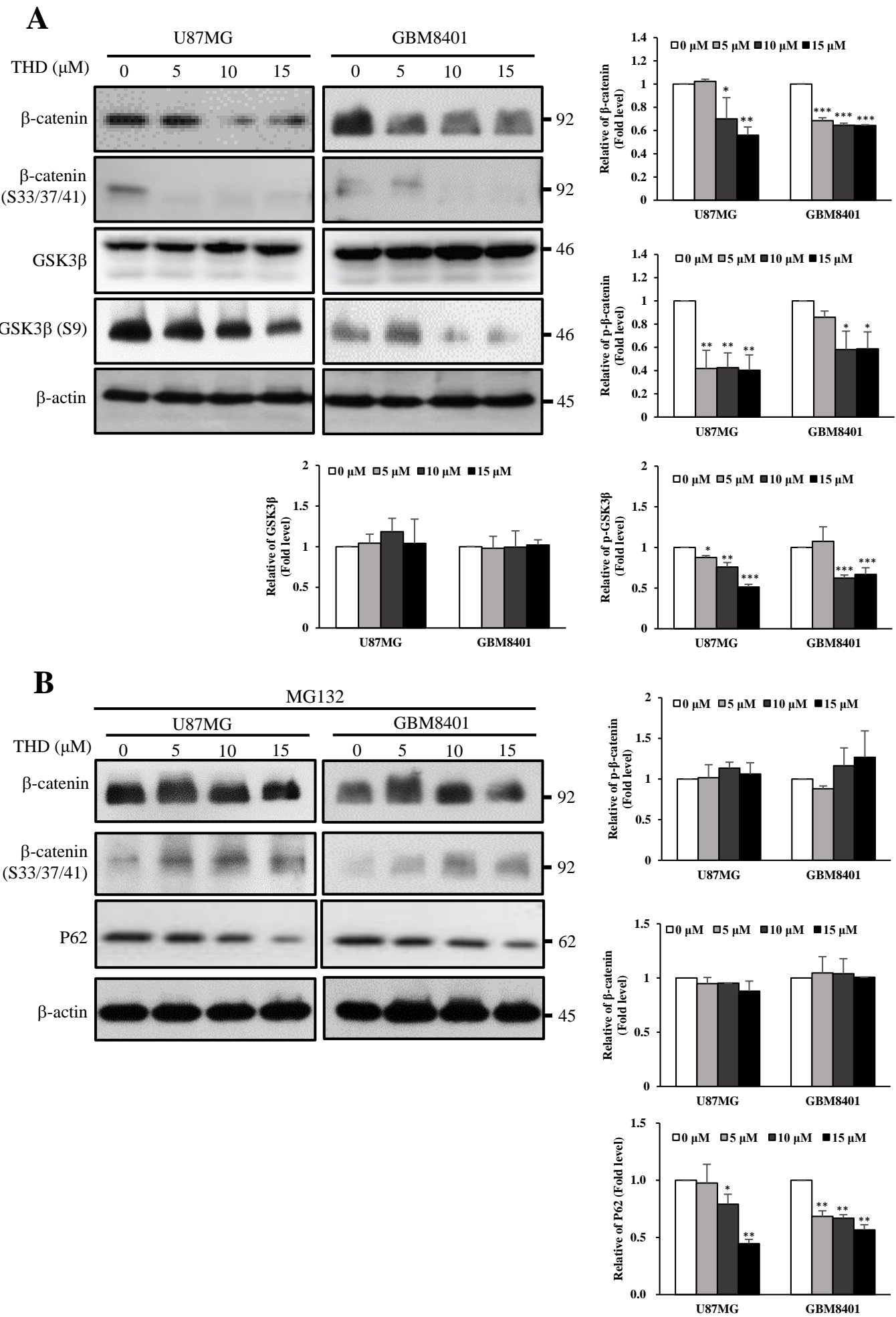

Supplement: Supplementary file 1 [file ijms-20-00473-s001.zip › PDF/Figure 5 Big.pdf]

**Fig 6**

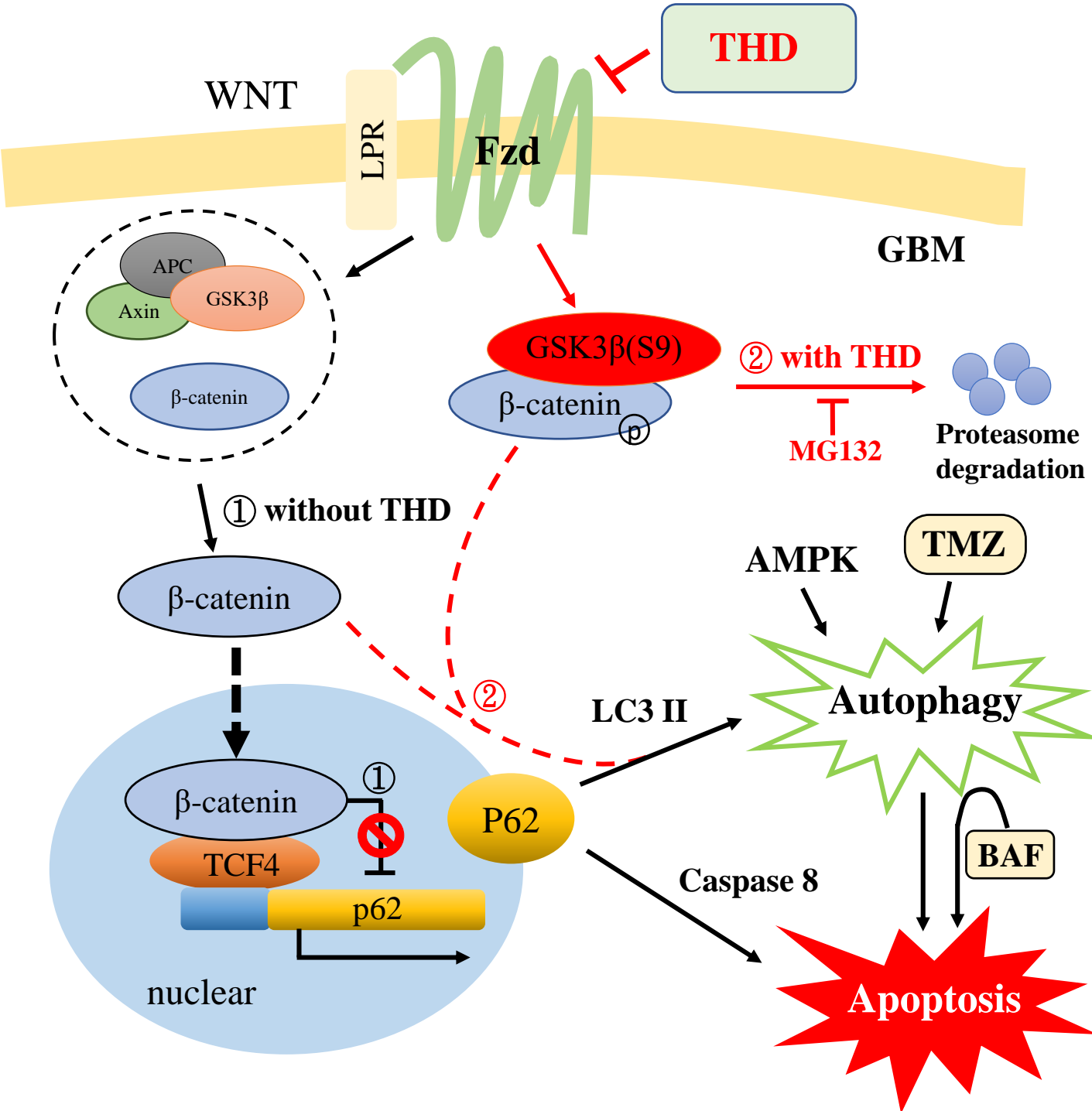

Supplement: Supplementary file 1 [file ijms-20-00473-s001.zip › PDF/Figure 6 Big.pdf]
